# Adjunctive posterior wall isolation for the treatment of persistent and longstanding persistent atrial fibrillation (CORNERSTONE AF) trial: Design and rationale

**DOI:** 10.1002/clc.24164

**Published:** 2023-10-11

**Authors:** Takatoshi Shigeta, Shinsuke Miyazaki, Osamu Inaba, Yukihiro Inamura, Junichi Nitta, Yukio Sekiguchi, Atsushi Takahashi, Hitoshi Hachiya, Yasutoshi Nagata, Yasuteru Yamauchi, Tatsuya Hayashi, Shinsuke Iwai, Akira Mizukami, Yuichi Ono, Keita Handa, Makoto Suzuki, Atsushi Suzuki, Jun Nakajima, Kenzo Hirao, Hiroyuki Okada, Miho Negishi, Takashi Ikenouchi, Tasuku Yamamoto, Kentaro Goto, Takuro Nishimura, Susumu Tao, Masateru Takigawa, Akihiro Hirakawa, Masahiko Goya, Tetsuo Sasano

**Affiliations:** ^1^ Department of Cardiovascular Medicine Tokyo Medical and Dental University Tokyo Japan; ^2^ Department of Cardiology Japanese Red Cross Saitama Hospital Saitama Japan; ^3^ Department of Cardiology Sakakibara Heart Institute Tokyo Japan; ^4^ Department of Cardiology Yokosuka Kyosai Hospital Kanagawa Japan; ^5^ Cardiovascular Center Tsuchiura Kyodo Hospital Ibaraki Japan; ^6^ Department of Cardiology Japanese Red Cross Musashino Hospital Tokyo Japan; ^7^ Department of Cardiology Japanese Red Cross Yokohama City Bay Hospital Kanagawa Japan; ^8^ Division of Cardiovascular Medicine, Saitama Medical Center Jichi Medical University Saitama Japan; ^9^ Department of Cardiology Hiratsuka Kyosai Hospital Kanagawa Japan; ^10^ Department of Cardiology Kameda Medical Center Chiba Japan; ^11^ Department of Cardiology Ome Municipal General Hospital Tokyo Japan; ^12^ Division of Cardiology Kashiwa City Hospital Chiba Japan; ^13^ Department of Cardiology Yokohama Minami Kyosai Hospital Yokohama Japan; ^14^ Heart Center Tokyo Yamate Medical Center Tokyo Japan; ^15^ Department of Cardiology Tokyo Metropolitan Toshima Hospital Tokyo Japan; ^16^ Arrhythmia Advanced Therapy Center AOI Universal Hospital Kanagawa Japan; ^17^ Department of Cardiology Soka Municipal Hospital Saitama Japan; ^18^ Department of Clinical Biostatistics, Graduate School of Medical and Dental Sciences Tokyo Medical and Dental University Tokyo Japan

**Keywords:** atrial fibrillation, catheter ablation, complication, posterior, pulmonary vein isolation, wall isolation

## Abstract

**Background:**

A left atrial posterior wall isolation (LAPWI) is one of the atrial fibrillation (AF) ablation strategies.

**Hypothesis:**

We hypothesized that an additional empirical LAPWI would increase the freedom from recurrent atrial arrhythmias as compared to standard AF ablation in persistent AF patients.

**Methods:**

The CORNERSTONE AF study is a prospective, randomized, multicenter study investigating patients with AF persisting for >7 days and <3 years undergoing first‐time AF ablation. They will be randomized to pulmonary vein isolation (PVI) or PVI + LAPWI in a 1:1 manner. Although PVI can be performed with either radiofrequency catheters or cryoballoons, only radiofrequency catheters will be permitted to achieve LAPWIs. Additional focal ablation targeting non‐pulmonary vein triggers will be allowed. A total of 516 patients will be enrolled in 17 centers between August 2022 and February 2024 based on the calculation with 80% power, considering the assumption that 65% and 75% of the PVI and PVI + LAPWI group patients will be free from atrial arrhythmia recurrence 18‐months postprocedure (10% of dropout). The primary endpoint is freedom from documented atrial arrhythmias 18 months postsingle procedures. Clinical follow‐up will include 7‐day ambulatory electrocardiograms and routine outpatient consultations by electrophysiologists at 1, 3, 6, 9, 12, and 18 months postprocedure.

**Results:**

As of August 2023, a total of 331 patients (68 ± 9 years, 270 men, 43 longstanding persistent AF) have been enrolled.

**Conclusions:**

The CORNERSTONE AF study is a prospective, randomized, multicenter trial designed to evaluate the efficacy and safety of an adjunctive empirical LAPWI following standard AF ablation in persistent AF patients.

## Graphical abstract

Patients with persistent AF or longstanding persistent AF will be randomized to a PVI alone or a PVI + LAPWI in a 1:1 manner. The PVI will be performed either by a radiofrequency catheter or cryoballoon. The left images show sample 3D mapping images after the procedure. AF, atrial fibrillation; LAPWI, left atrial posterior wall isolation; PVI, pulmonary vein isolation.

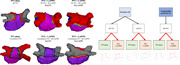

## 1 INTRODUCTION

Catheter ablation is an important therapeutic strategy for rhythm control of atrial fibrillation (AF) and its cornerstone is pulmonary vein isolation (PVI).^1^ In fact, although there are various approaches for treating AF, the STAR AF Ⅱ trial failed to prove any additional efficacy of linear ablation or complex fractionated electrograms ablation compared to a PVI alone.^2^ However, in persistent AF (PsAF) patients, a sufficient clinical outcome could not be obtained by a PVI alone, considering the result that 40% of patients were suffering from atrial arrhythmias recurrences after a single ablation procedure in STAR AF Ⅱ. One of the possible substrates beyond the pulmonary veins (PVs) is the left atrial posterior wall (LAPW). The LAPW is included in the PV component of the left atrium (LA) and its smooth tissue differs embryologically from the other primitive cardiac tube.^3^ The various distributions of the myocardial architecture can produce nonuniform anisotropic conduction leading to arrhythmogenicity.

The LAPW isolation (LAPWI), so called “box isolation,” is a strategy targeting the arrhythmogenicity of the PV component, which is achieved by linear ablation of the roof and floor of the LA connecting both PVI circles in addition to the PVI. Although a meta‐analysis including 11 studies showed a lower atrial arrhythmia recurrence rate after an adjunctive LAPWI as compared with a PVI alone in PsAF or longstanding PsAF (long‐PsAF) patients, the analysis included studies in which the recurrence rate was higher in the LAPWI group.^4^ Among the randomized controlled trials (RCTs) included in the meta‐analysis, two RCTs failed to prove any superiority of the LAPWI to a PVI alone.^4^, ^5^ Further, two RCTs favorable for an LAPWI in the meta‐analysis were not considered to be randomized studies for the aim of comparing PVI alone and an additional LAPWI.^6^, ^7^ The evidence of an empiric LAPWI in PsAF/long‐PsAF patients has not been established sufficiently yet.

On the other hand, triggers other than the PVs (non‐PV triggers), which initiate AF, have been focused on as a strategy for AF ablation. It has been reported that non‐PV triggers are identified in ~10% of PsAF/long‐PsAF patients.^8^ In another investigation, sufficient clinical outcomes could be obtained in those with treatable non‐PV triggers as compared with those with persistent triggers after ablation for non‐PV triggers such as including a superior vena cava (SVC) isolation or LAPWI.^9^ According to the expert consensus statement, it is recommended to consider ablation of reproducible focal triggers identified outside the PV ostia (Class Ⅱa), which is more strongly recommended than an LAPWI (Class Ⅱb).^10^ However, this ablation strategy was not adopted in most of the trials in which the efficacy of an additional LAPWI was examined. Therefore, there is a value in evaluating the true efficacy of an additional empirical LAPWI after a conceivable best practice of AF ablation including a non‐PV trigger ablation in addition to the PVI in PsAF/long‐PsAF patients.

With regard to the safety, possible gastroesophageal complications need to be considered, especially when an LAPWI is added because the PV component is closely adjacent to the esophagus and periesophageal vagal nerve branches. There are some investigations of esophageal thermal injury including cases in which linear ablation of the roof or floor of the LA was performed, but they showed that linear ablation was not associated with increasing esophageal injuries.^11^, ^12^ Some observational studies reported that the prevalence of gastric hypomotility detected on gastroesophageal endoscopy, which is attributed to the periesophageal vagal nerve injury, was significantly higher in cases with an additional LAPWI as compared to those with a PVI alone.^13^, ^14^ Further investigations regarding the safety after an additional LAPWI are required.

The incidence of iatrogenic atrial tachycardia (AT) after the procedure would be a potential disadvantage of an adjunctive LAPWI. According to the meta‐analysis of LAPWIs, the prevalence of an AT recurrence tends to be higher after an additional LAPWI than after a PVI alone.^4^ In addition, there has been a report that demonstrated that peri‐mitral ATs were the main cause of recurrent ATs^15^ after an LAPWI. The influence of an additional LAPWI should be investigated further.

We planned this prospective RCT of the CORNERSTONE AF study to prove the efficacy of an empiric LAPWI in addition to the standard AF ablation in PsAF/long‐PsAF patients. We hypothesized that an additional empirical LAPWI would increase the freedom from recurrent atrial arrhythmias as compared to standard AF ablation in PsAF/long‐PsAF patients. We also aim to investigate the details of the procedure with regard to not only the efficacy but also the safety including gastric hypomotility.

## 2 METHODS

### 2.1 Study design

**Figure 1:**
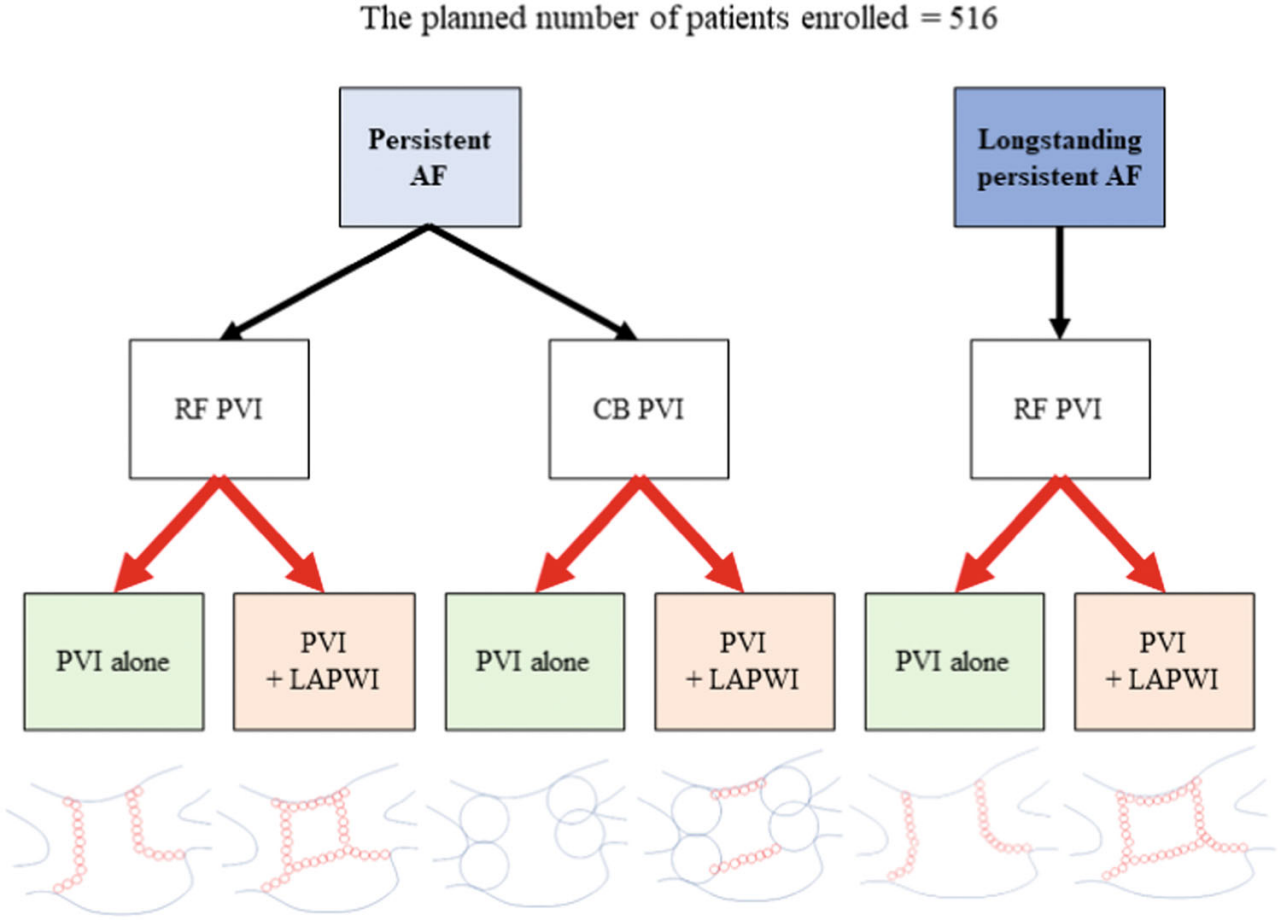
Randomization details. For patients with persistent AF (PsAF), it will be allowed to perform the PVI by either a radiofrequency catheter or CB. Note that the operators should choose what they will use for the PVI before randomization. After the decision, randomization will be implemented before the procedure. Regarding longstanding PsAF (long‐PsAF), only a radiofrequency catheter will be permitted to be utilized for the PVI. Allocation of the patients to a PVI alone or a PVI + LAPWI will be performed in a 1:1 manner in each group, namely those with PsAF, who will undergo a PVI with a radiofrequency catheter; those with PsAF, who will undergo a PVI with a CB; and those with long‐PsAF (shown as red arrows). The large blue circles indicate CB ablation lesions and small red circles indicate radiofrequency ablation lesions. AF, atrial fibrillation; AFL, atrial flutter; AT, atrial tachycardia; CB, cryoballoon; LAPWI, left atrial posterior wall isolation; PVI, pulmonary vein isolation; RF, radiofrequency.

The CORNERSTONE AF study is a prospective, multicenter, randomized, open‐label trial performed at 17 centers in Japan (coordinating center: Tokyo Medical and Dental University, principal investigator: Tetsuo Sasano, details in Supporting Information: Appendix), aiming to assess the efficacy of empirical LAPWI in addition to standard AF ablation in PsAF/long‐PsAF patients. AF is classified according to the latest guidelines.^1^ The patients with AF lasting for more than 7 days and less than 3 years, who undergo a de novo AF ablation, will be enrolled and centrally allocated to a PVI alone or PVI + LAPWI in a 1:1 manner before the ablation procedure using a permuted block randomization. The duration of AF will be defined as the persistence of the AF. Patients with paroxysmal AF will not be enrolled. Randomization will be stratified by the institution, type of AF (i.e., PsAF or long‐PsAF), and kind of instrument utilized for the PVI (i.e., radiofrequency catheter or cryoballoon) (Figure 1). The outcome of the randomization will not be blinded to either the operators, the patients or the attending physicians. No funding was used to support this work. This study has been registered at UMIN (University hospital Medical Information Network) Clinical Trials Registry (UMIN 000047638, https://center6.umin.ac.jp/cgi-open-bin/ctr_e/ctr_view.cgi?recptno=R000054321) and has been approved by the institutional review board at each participating hospital. A signed, institutional review board‐approved informed consent form will be obtained from each patient before enrollment.

### 2.2 Endpoints

The primary endpoint is designed as the freedom from atrial arrhythmia recurrence such as AF, AT, and atrial flutter (AFL) with or without antiarrhythmic drugs at 18 months after a single procedure. Atrial arrhythmia recurrence is defined as documented AF/AT/AFL lasting more than 30 s after a 90‐day blanking period according to the guidelines.^1^ The safety endpoints include the incidence of procedure‐related major complications such as a stroke, transient ischemic attack, PV stenosis, cardiac tamponade, pericarditis, phrenic nerve injury, major bleeding, vascular access complications (significant groin hematoma, pseudoaneurysm, arteriovenous fistula), atrioesophageal fistulae, gastric hypomotility, heart failure, myocardial infarction, and death according to the guidelines.^1^ To ensure the safety, all adverse events will be reported by the investigators to the coordinating center and reviewed by the institutional review board every month (the members are listed in the Supporting Information: Appendix). In addition, data monitoring will be performed every 6 months by two cardiologists independent of the present study.

The secondary endpoints include the freedom from individual components of the primary outcome (AF, AT, and AFL), atrial arrhythmia freedom after multiple procedures, atrial arrhythmia burden reduction, recurrent arrhythmia types (paroxysmal or persistent), symptomatic atrial arrhythmia recurrence free rate, comparison regarding the atrial arrhythmia freedoms between the radiofrequency group and cryoballoon group in PsAF patients, direct‐current cardioversion free rate, procedure time and fluoroscopy time, success rate of the LAPWI, dimension of the additional isolated area of the LAPW after the LAPWI, freedom from re‐do procedures, durability of the PVI and LAPWI during the second procedure among the patients who underwent a redo procedure, number of repeat procedures, and gastrointestinal symptoms during the periprocedural period. The predictors of atrial arrhythmia recurrence will be also examined among the various factors including the existence of low voltage areas and the kind of devices such as the three‐dimensional mapping systems and ablation catheters.

### 2.3 Study patients

All criteria for inclusion and exclusion are presented in Table 1. All patients must exhibit an episode of continuous AF that is sustained >7 days in the year before the procedure. Exclusion criteria will be as follows: (1) patients with AF lasting >3 years; (2) patients aged <20 years old; (3) patients with a history of previous cardiac surgery; (4) patients with an LA diameter of >55 mm measured in the parasternal view on trans‐thoracic echocardiography; (5) patients with heart failure with a New York Heart Association class Ⅳ; (6) patients with hemodialysis; and (7) patients presumed to require the use of amiodarone after the procedure such as those with ventricular tachycardia. The other criteria are described in Table 1. Patients with a history of a previous cavotricuspid isthmus ablation or paroxysmal supraventricular tachycardia ablation will not be excluded.

**Table 1:** Criteria for inclusion and exclusion.

Inclusion
Patients with PsAF (lasting >7 days)
Patients undergoing a de novo AF ablation
Patients expected to fulfill an 18‐month follow‐up after the procedure
Exclusion
Patients with AF lasting >3 years
Patients aged <20‐year‐old
Patients with a history of previous cardiac surgery
Patients with an LAD > 55 mm
Patients with heart failure with NYHA class Ⅳ
Patients with a reversible cause such as hyperthyroidism
Patients with contraindications to anticoagulants
Patients with intracardiac thrombi
Pregnant patients
Patients with hemodialysis
Patients presumed to require the use of amiodarone after the procedure

Abbreviations: AF, atrial fibrillation; LAD, left atrial diameter; PsAF, persistent AF; NYHA, New York Heart Association.

### 2.4 Ablation procedure

**Figure 2:**
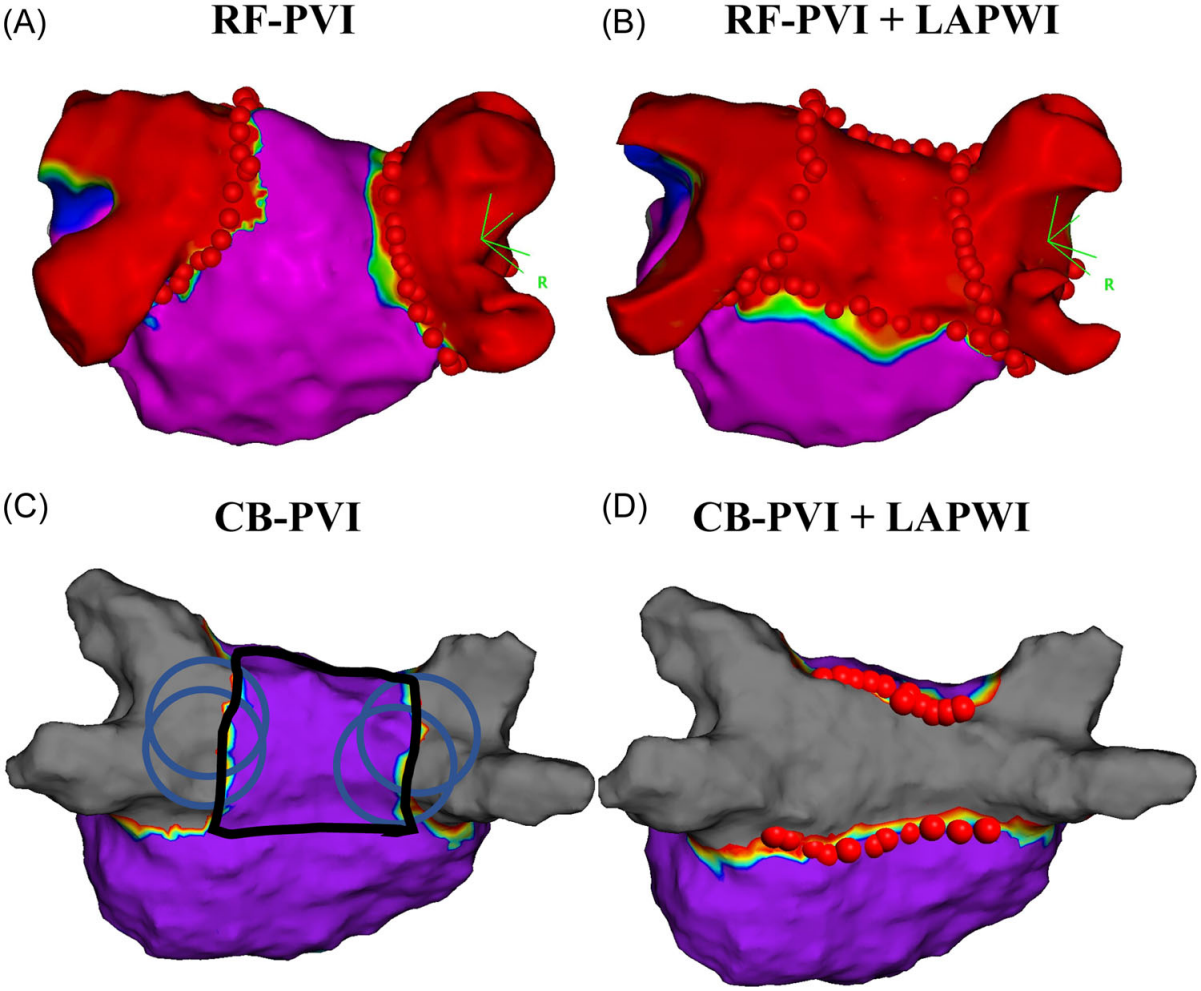
Ablation procedure details. (A, B) Posterior–anterior view of the voltage map (CARTO) during CS pacing after the PVI (A) and LAPWI (B) with a radiofrequency catheter in the same case. (C, D) Posterior–anterior view of the voltage map (EnsiteⅩ) during CS pacing after the PVI with a CB (C) and LAPWI with a radiofrequency catheter (D) in the same case. In each figure, the blue circles are depicted as the estimated positions of the CB, the ablation points are described as red‐colored tags, and the purple area indicates a voltage ≥0.5 mV. In the EnsiteⅩ voltage maps, the gray area indicates a voltage ≤0.1 mV, and in the CARTO voltage maps, the red area indicates a voltage ≤0.1 mV. The isolated area shown as that surrounded by the black line in each figure will be measured. CB, cryoballoon; CS, coronary sinus; LAPWI, left atrial posterior wall isolation; PVI, pulmonary vein isolation; RF, radiofrequency.

The details of the ablation procedure such as the vascular approach, anesthesia method, use of an esophageal temperature probe, or choice of the 3‐dimensional mapping system will be left to each institution's discretion. Uninterrupted oral anticoagulants will be generally required. First, a PVI will be performed utilizing either a radiofrequency catheter or cryoballoon in PsAF patients and will be performed with a radiofrequency catheter in long‐PsAF patients. In the PsAF patients, the choice of radiofrequency catheter or cryoballoon will be determined before the randomization (Figure 1). This is because cryoballoon use has been approved for a PVI alone in PsAF patients but not in long‐PsAF patients in Japan. All the ablation procedures can be performed during any rhythm (AF, sinus rhythm, and so on). After the confirmation of the electrical PVI, a voltage map of the LA will be created during sinus rhythm or atrial pacing rhythm, if sinus rhythm can be maintained. In the patients randomized to the LAPWI group, linear ablation of the LA roof and floor will be performed with a radiofrequency catheter (Figure 2). The endpoint of the LAPWI is defined as no near‐field potentials and no atrial capture when pacing is performed from inside the “box” with the ablation catheter with more than 5 V and a 2 ms output. If an LAPWI is not achieved after the LA roof and floor linear ablation, additional focal ablation of the LA posterior wall (inside the “box”) will be allowed. After the accomplishment of the LAPWI, a voltage map of the LA will be created again to assess the additional isolated area. In addition to the PVI or LAPWI, a cavotricuspid isthmus ablation and SVC isolation will not be prohibited. When non‐PV foci, which is defined as ones cause AF initiation or frequent atrial ectopy, will be identified by an isoproterenol infusion and/or electrical cardioversion, focal ablation will also be permitted. However, no other empirical ablation including linear ablation (mitral isthmus ablation, and so on), low voltage area ablation, electrogram based ablation, or vein of Marshall ethanol infusion will be allowed. In the patients randomized to the PVI group, an LAPWI will be considered only when the multiple triggers identified on the LAPW cannot be controlled by focal ablation. Otherwise, an LAPWI will not be permitted.

### 2.5 Postablation management

**Figure 3:**
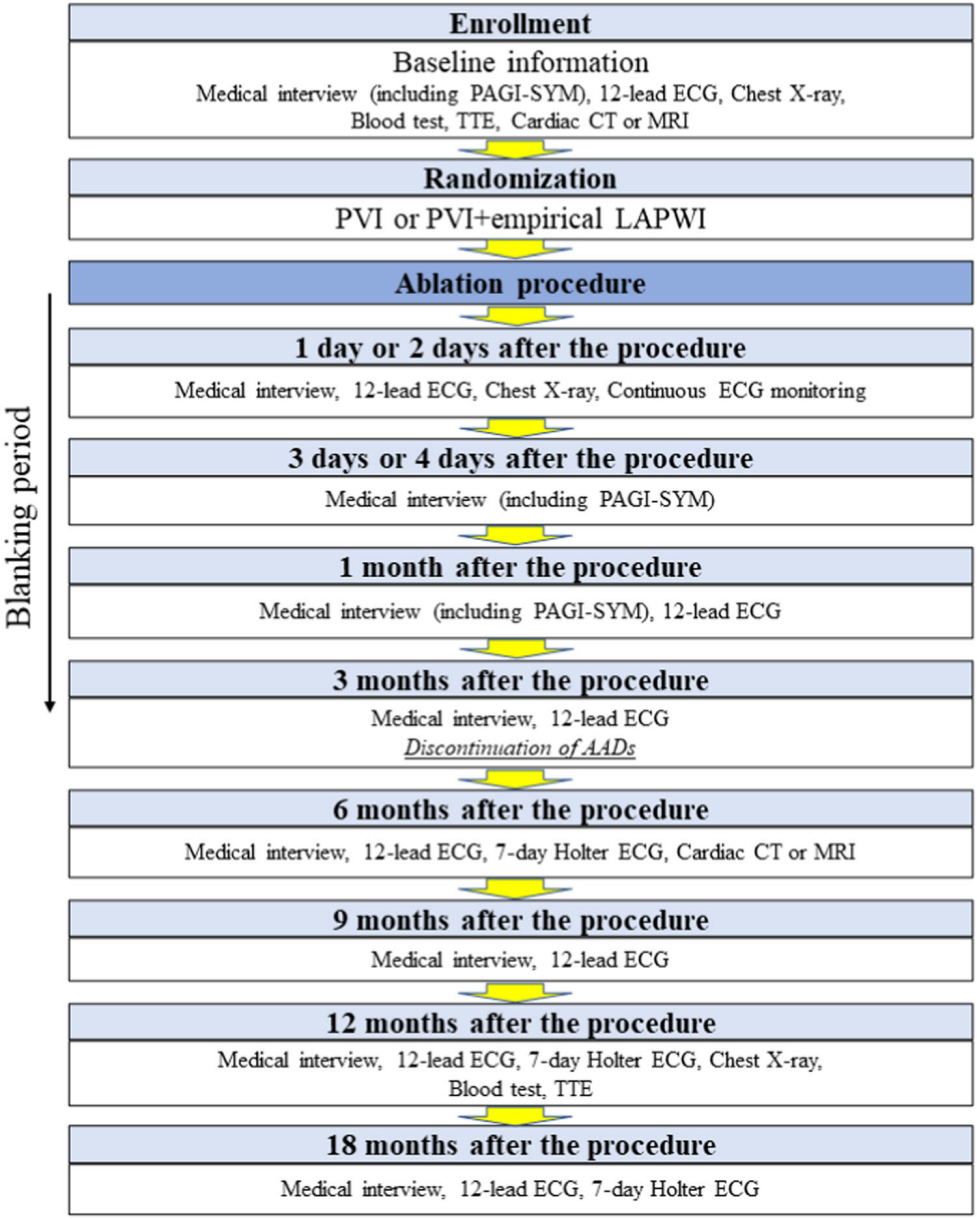
CORNERSTONE AF trial flow chart. AADs, antiarrhythmic drugs; CT, computed tomography; ECG, electrocardiography; LAPWI, left atrial posterior wall isolation; MRI, magnetic resonance imaging; PAGI‐SYM, patient assessment of upper gastrointestinal symptom severity index; PVI, pulmonary vein isolation; TTE, trans‐thoracic echocardiography.

All patients will receive a proton pump inhibitor for at least 1 month. Follow‐up visits will be required at 1, 3, 6, 9, 12, and 18 months after the ablation procedure in all patients, and they will undergo 12‐lead electrocardiograms at each visit (Figure 3). Routine 7‐day ambulatory electrocardiograms (7‐day Holter ECG) (Gram, Durantis Co. Ltd) will be performed at 6, 12, and 18 months after the procedure, and analyzed by the staff who are not involved with the study outcomes. The electrophysiologists will evaluate the clinical outcomes at every visit in person, and additional monitoring such as, 24 hours to 7 day Holter electrocardiograms, patient activated event recorders for 1 month (HCG 801, Omron), 7‐ to 14‐day autotriggered event recorders (SpiderFlash, ELA Medical), or commercially available photoplethysmography (Apple Watch, Apple Inc.) will be used if necessary.

Antiarrhythmic drugs will be basically discontinued within 3 months after the procedure. Amiodarone use will not be permitted after the procedure. Trans‐thoracic echocardiography will be performed before the procedure and at 12 months after the procedure to assess the atrial remodeling and cardiac function. Cardiac enhanced computed tomography will also be considered before the procedure and at 6 months after the procedure, if the patients consent to receive it, to examine whether there will be PV stenosis. To evaluate the symptomatic gastric hypomotility, the patients will be required to report their symptoms through a 20‐question Patient Assessment of Upper Gastrointestinal Disorders‐Symptom Severity Index^16^ before the procedure, and 3–4 days and 1 month after the procedure. The responses to each question in this tool will be rated from 0 to 5, with a score of 5 indicating a severe symptom. Redo procedures will be allowed at any time after the initial ablation procedure according to the physicians' decision if patients suffer from any atrial arrhythmia recurrence, and the strategy of the procedure will be left to the operators' discretion. The durability of the PVI and LAPWI will be evaluated only in patients who undergo redo procedures.

### 2.6 Sample size calculation

The sample size is based on the primary endpoint of this study, namely the atrial arrhythmia recurrence‐free rate at 18 months after a single procedure. Based on the previous literature,^2^, ^17^ we assume that 65% of PsAF/long‐PsAF patients will be free from atrial arrhythmia recurrence at 18 months after a PVI alone and that the atrial arrhythmia free rate will increase by 10% when an LAPWI is additionally performed. Moreover, we assume the possibility of a 10% dropout of participants. To reject the null hypothesis that the probability of the primary endpoint will be the same between the experimental and control subjects, with a power of 80% and a significant level of 5% (two‐sided), a total of 516 patients will be required with a randomization ratio of 1:1.

### 2.7 Statistical analysis

The full analysis set (FAS) for the primary efficacy analysis is defined as the set of all randomized patients who meet all inclusion criteria and its statistical evaluation is based on the intention‐to‐treat principle. An analysis of the per‐protocol set will be also be performed to assess the robustness of the results of the analysis using FAS. Continuous variables of the baseline patient characteristics will be expressed as the mean ± SD, or median with the interquartile range, and their distributions among the groups will be compared utilizing a Student *t* test or Mann‐Whitney *U* test. Categorical variables of the baseline patient characteristics will be reported as numbers and percentages, and their distributions among the groups will be compared with the *χ* ^2^ or Fisher exact tests. Missing data will not be imputed in these analyses.

The survival curves of the primary endpoint will be estimated using the Kaplan–Meier method and the difference in them between the additional LAPWI group and PVI alone group will be compared based on the log‐rank test. Patients not reported as having atrial arrhythmia recurrence events at 18 months and patients lost to follow‐up within 18 months will be censored at 18 months and the date they are last known to be free from any atrial arrhythmia recurrence, respectively.

No interim analyses will be conducted in this trial. A *p* < .05 will be considered to indicate statistical significance. A Cox regression analysis will also be utilized for the multivariate analysis regarding the freedom of atrial arrhythmia recurrence. For the secondary endpoints, the AF recurrence‐free rate and AT recurrence‐free rate will be analyzed with the same method as the primary endpoint. The AF burden reduction, total number of ablation sessions, and complication rate will be analyzed between the two groups by an appropriate statistical test according to the type of variables. There are no prespecified subanalyses.

## 3 RESULTS

### 3.1 Study patients

The first patient was enrolled on August 4, 2022, and the enrollment of patients will continue until February 2024. As of August 2023, a total of 331 patients were enrolled. The mean age was 68 ± 9 years old, 270 (81.6%) were men, and 288 (87.0%) patients had persistent AF (Table 2).

**Table 2:** Preliminary data for patients enrolled as of August 2023.

	N = 331
Age, years	68 ± 9
Male gender, *n* (%)	270 (81.6%)
Body mass index	24.7 ± 3.8
PsAF, *n* (%)	288 (87.0%)
Left atrial diameter, mm	41.8 ± 5.9
Left ventricular ejection fraction, %	59.7 ± 10.4

Abbreviation: PsAF, persistent AF.

## 4 DISCUSSION

An LAPWI is an option as an adjunctive therapeutic strategy after a PVI in PsAF/long‐PsAF patients. However, it is controversial to perform an LAPWI empirically, which is reflected in the current guidelines of AF (Class Ⅱb).^1^ One of the reasons seems to be a lack of sufficient evidence regarding its efficacy. A recent meta‐analysis demonstrated that the prevalence of atrial arrhythmia recurrence is significantly lower in patients who undergo an additional LAPWI as compared to those who undergo a PVI alone.^4^ However, it included 2 RCTs in which there was no significant difference in the arrhythmia freedom rate between the adjunctive LAPWI group and PVI alone group.^4^, ^5^ In one of them, the number of study patients was small and the percentage of patients who continued antiarrhythmic medications after the procedure was relatively high. Further, reconnections of the LAPWI were confirmed during the repeat ablation in all patients.^4^ Moreover, previous RCTs showed that the atrial arrhythmias recurrence rate is not significantly lower after an additional LAPWI as compared with after a PVI alone; however, those trials included patients with paroxysmal AF or those who underwent an MI ablation.^18^, ^19^ According to another meta‐analysis including more previous studies regarding an LAPWI, the reconnection rate of an LAPWI was relatively high.^20^ It might be speculated that a poor lesion durability of the LAPW might contribute to no additional impact on the clinical outcomes. The recent published randomized trial, CAPLA, failed to prove the benefit of the empirical LAPWI in PsAF patients.^21^ However, the success rate of LAPWI was 86.5% and focal ablations within LAPW was required in 50.3% of patients; moreover, the reconnection of LAPWI was confirmed in 68.8% of patients who underwent redo ablation in the trial.^21^ Considering these results, there are still a lot to be investigated in empirical LAPWI.

There are several important differences in the present study design from the previous trials investigating the impact of an additional LAPWI. First, targeting non‐PV AF triggers will be allowed in the present study. The efficacy of targeting reproducible focal triggers identified outside the PV ostia has been established and recommended in the guidelines (Class Ⅱa). The present study for the first time will provide data on whether an additional empirical LAPWI will further improve the arrhythmia freedom rate beyond the current standard AF ablation strategy. Second, in contrast to the previous RCTs, cryoballoon use will be allowed for the PVI in PsAF patients in line with current clinical practice. The efficacy and safety of the cryoballoon PVI has been well established, and the cryoballoon is currently widely used in clinical practice. Although a small study suggested that an additional LAPWI with the cryoballoon had a beneficial effect on AF recurrence as compared to a cryoballoon PVI alone,^22^ the results need to be validated in ongoing trial.^23^ Third, the present study will not exclude asymptomatic patients, antiarrhythmic drug naïve patients, or patients with antiarrhythmic drug effective AF, to reflect the clinical practice of these days. The symptoms in patients with PsAF/long‐PsAF are often unclear; however, AF ablation is chosen as a treatment option in those populations in the clinical practice. Moreover, the clinical benefit of an early, systematic rhythm control does not differ between asymptomatic and symptomatic patients as shown in EAST‐AFNET 4.^24^ Fourth, although the low durability of the LAPWI and risk of iatrogenic ATs after the LAPWI have been reported, the higher durability will be expected in the present study owing to the rapid progress of current ablation devices and technologies. Fifth, the present study will evaluate the peri‐esophageal vagal nerve injury using a specific questionnaire.^16^ Symptomatic gastric hypomotility is a rare complication; however, previous studies suggested that an additional LAPWI increases the incidence of endoscopy‐detected silent gastric hypomotility after the procedure.^13^, ^14^ As the symptoms are generally exacerbated by a full stomach, 3–4 days after the procedure would be the appropriate timing to evaluate the symptoms. Therefore, the present study will provide various valuable evidence regarding an additional LAPWI in the current standard AF ablation in patients with PsAF/long‐PsAF.

### 4.1 Limitations

There are several limitations to be mentioned. First, an additional focal ablation of non‐PV triggers will be permitted, therefore, we cannot compare the outcomes between a PVI alone and a PVI + LAPWI as in the CAPLA trial. However, we aim to evaluate the impact of an empirical LAPWI in addition to standard AF ablation, which consists of a PVI (Class Ⅰ indication) and non‐PV trigger ablation (Class Ⅱa indication). Second, the randomization will not be blinded to the patients and physicians during the follow‐up, or there will be no blinded committee to assess the endpoint, which might produce some bias, however, the results of 7‐day Holter ECG recordings will be analyzed by staff blinded to the randomization. Third, the PVI will be performed with either a cryoballoon or radiofrequency catheter, which will produce an additional variable. However, we aim to assess the impact of an additional LAPWI in the clinical practice. Considering that the cryoballoon is widely utilized for the PVI, even for PsAF, it is reasonable to include cryoballoon ablation. Fourth, there will be no committee that will oversee the efficacy and safety of the study. However, to ensure the safety, all adverse events will be reported to the coordinating center, and the institutional review board will assess whether there is any issue to continue the study every month (member list in Supporting Information: Appendix). In addition, data monitoring will be performed every 6 months by 2 cardiologists independent of this study (member list in Supporting Information: Appendix). Fifth, although the procedural endpoint has been clearly determined, the detailed ablation protocol to reach the endpoint is left to each operator.

## 5 CONCLUSIONS

The CORNERSTONE AF study is a prospective, randomized, multicenter trial designed to evaluate the efficacy and safety of an adjunctive empirical LAPWI following standard AF ablation in PsAF patients.

## CONFLICT OF INTEREST STATEMENT

The authors declare no conflict of interest.

## Supporting information

Supporting Information.

## Data Availability

The data that support the findings of this study are available from the corresponding author upon reasonable request.
