# Supplementary material for: Adjunctive posterior wall isolation for the treatment of persistent and longstanding persistent atrial fibrillation (CORNERSTONE AF) trial: Design and rationale
Source: Clin Cardiol. 2023 Oct 11;47(1):e24164. doi: 10.1002/clc.24164 (PMC10766127; doi:10.1002/clc.24164)
Supplement: Supplementary file 1 — Supporting Information. [file CLC-47-e24164-s001.docx]

Institute, Principal investigator (PI)

1. Tokyo Medical and Dental University, Tokyo, Japan (Coordinating center)

PI: Tetsuo Sasano

1. Japanese Red Cross Saitama Hospital, Saitama, Japan

PI: Osamu Inaba

1. Sakakibara Heart Institute, Tokyo, Japan

PI: Junichi Nitta

1. Yokosuka Kyosai Hospital, Kanagawa, Japan

PI: Atsushi Takahashi

1. Tsuchiura Kyodo Hospital, Ibaraki, Japan

PI: Hitoshi Hachiya

1. Japanese Red Cross Musashino Hospital, Tokyo, Japan

PI: Yasutoshi Nagata

1. Japanese Red Cross Yokohama City Bay Hospital, Kanagawa, Japan

PI: Yasuteru Yamauchi

1. Saitama Medical Center, Jichi Medical University, Saitama, Japan

PI: Tatsuya Hayashi

1. Hiratsuka Kyosai Hospital, Kanagawa, Japan

PI: Shinsuke Iwai

1. Kameda Medical Center, Chiba, Japan

PI: Akira Mizukami

1. Ome Municipal General Hospital, Tokyo, Japan

PI: Yuichi Ono

1. Kashiwa City Hospital, Chiba, Japan

PI: Keita Handa

1. Yokohama Minami Kyosai Hospital, Yokohama, Japan

PI: Makoto Suzuki

1. Tokyo Yamate Medical Center, Tokyo, Japan

PI: Atsushi Suzuki

1. Tokyo Metropolitan Toshima Hospital, Tokyo, Japan

PI: Jun Nakajima

1. AOI Universal Hospital, Kanagawa, Japan

PI: Kenzo Hirao

1. Soka Municipal Hospital, Saitama, Japan

PI: Hiroyuki Okada

Institutional review board (IRB) member in Tokyo Medical and Dental University

1. Hiromitsu Takahashi
2. Yusuke Ebana
3. Hiroyuki Kagechika
4. Kazuhisa Hasebe
5. Takehiko Mori
6. Emiko Asaka
7. Naoko Harada
8. Yoshiki Tanaka
9. Masako Ishibashi
10. Toshiko Saito
11. Junko Sekiguchi
12. Kaoru Hirota

Data monitoring

1. Yuji Matsuda, MD.
2. Kensuke Hirasawa, MD.
